# Gender differences in response to cold pressor test assessed with velocity-encoded cine-MR imaging of the coronary sinus

**DOI:** 10.1186/1532-429X-13-S1-P346

**Published:** 2011-02-02

**Authors:** Alexis Jacquier, Antonin Flavian, Frank Kober, Francesca Carta, Boris Maurel, Patrick J Cozzone, Monique Bernard

**Affiliations:** 1 CHU La Timone, CEMEREM, Marseille, France

## Introduction

Gender-specific differences in cardiovascular risk are well known, and current evidence supports an existing role of coronary endothelium function in these differences.

## Purpose

To assess non invasively coronary endothelial function in male and female young volunteers by myocardial blood flow (MBF) measurement using coronary sinus (CS) flow quantification by velocity encoded cine MRI at rest and during cold pressor test (CPT).

## Methods

Twenty-four healthy volunteers (12 men, 12 women) underwent MRI in a 3 Tesla scanner (Verio, Siemens, Erlangen, Germany). CPT was performed by immersing the right ankle in ice-water during 4 min. Heart rate and blood pressure were monitored throught the protocol. Coronary sinus flow was measured at rest and during CPT using a non breath-hold velocity encoded phase contrast cine MRI (TR/TE: 45 ms / 2 ms, slice thickness:5.5mm, FOV: 250^2^, averages:11, matrix:256^2^, flow encoding 70cm/s, flip angle: 25°, acquisition time: 4 min, GRAPPA k-space reduction factor:4). Left ventricular volumes, function and morphology were evaluated using SSFP sequence. MBF was calculated combining coronary sinus flow quantification and morphologic data using Argus work station (Siemens, Erlangen, Germany). Coronary endothelial function was assessed by comparing MBF at rest and during CPT. Coronary vascular resistance (CVR), rate pressure product and endothelium-dependent vasodilatation index (EDVI) were calculated.

## Results

**Figure 1:**
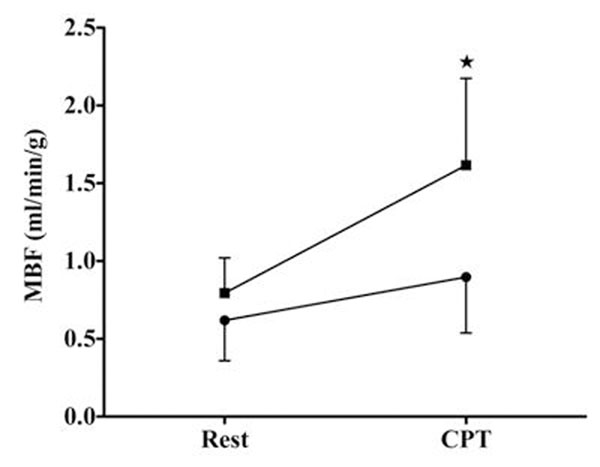
Comparison of myocardial blood flow (MBF) obtained at rest and after CPT in men (circle) and women (square).

At baseline, MBF was 0.63 ± 0.23 mL.g^-1^.min^-1^ in men and 0.79 ± 0.21 mL ·g^-1^ ·min^-1^ in women (P=ns) (figure 1). During CPT, the rate pressure product in men significantly increased by 49 ± 36 % (p<0.0001) and in women by 52 ± 22 % (p<0.0001). MBF increased significantly in both men and women by 0.22 ± 0.19 mL·g^-1^·min^-1^ (p=0.0022) and by 0.73 ± 0.43 mL·g^-1^·min^-1^ (p=0.0001), respectively. The increase in MBF was significantly higher in women than in men *(*p = 0.0012)

## Conclusions

MRI coronary sinus flow quantification for measuring myocardial blood flow revealed a higher response of MBF to CPT in women than in men. This finding may reflect gender differences in endothelial-dependent vasodilatation. This non invasive rest/stress protocol may become helpful to study endothelial function in normal physiology and in physiopathology.

